# Rapid formation of pseudoaneurysm of the mitral–aortic intervalvular fibrosa in a patient with bicuspid aortic valve and infective endocarditis: a case report

**DOI:** 10.1186/s13256-026-06047-2

**Published:** 2026-04-18

**Authors:** Lanhua Chen, Huizhong Li, Rong Chen, Yunhai Liao

**Affiliations:** 1 Department of Ultrasound Diagnosis, 900th Hospital of Joint Logistics Support Force of PLA, Fuzhou, Fujian China; 2 Department of Emergency, The Second Affiliated Hospital of Fujian University of Traditional Chinese Medicine, Fuzhou, Fujian China

**Keywords:** Pseudoaneurysm of the mitral–aortic intervalvular fibrosa, Infective endocarditis, Bicuspid aortic valve, Transesophageal echocardiography, Case report

## Abstract

**Background:**

Pseudoaneurysm of the mitral–aortic intervalvular fibrosa (P-MAIVF) is a rare yet severe cardiac pathology that typically arises as a complication of infective endocarditis or cardiac surgery. This condition is associated with a high mortality rate, particularly in instances where timely surgical intervention is not conducted. Transesophageal echocardiography (TEE) is the preferred diagnostic modality, offering detailed visualization of the pathological communication between the pseudoaneurysm and the left ventricular outflow tract, along with its dynamic changes throughout the cardiac cycle.

**Case presentation:**

A 28-year-old Han Chinese woman with a congenital bicuspid aortic valve (BAV) presented with recurrent fever suggestive of infective endocarditis (IE). Cardiac ultrasound revealed vegetations on the aortic valve along with a mitral–aortic intervalvular fibrosa pseudoaneurysm. Contrast-enhanced chest CT demonstrated a pseudoaneurysm communicating with the left ventricular outflow tract. The patient subsequently underwent aortic valve replacement and repair of the mitral–aortic fibrous connection under extracorporeal circulation. The patient recovered successfully with an uneventful postoperative course.

**Conclusion:**

This case underscores that congenital BAV is a significant predisposing factor for the rapid development of P-MAIVF in the setting of infective endocarditis. TEE is the diagnostic modality of choice for characterizing the lesion and its hemodynamic consequences. Early recognition and timely surgical intervention are crucial to prevent catastrophic rupture and ensure favorable outcomes.

## Introduction

P-MAIVF is a rare but potentially life-threatening cardiac condition, most frequently encountered as a complication of infective endocarditis (IE). Its key etiologies include infective endocarditis, surgical trauma, and congenital anomalies. The nonspecific clinical and imaging features of P-MAIVF often lead to misdiagnosis, such as aortic root abscesses, intra-atrial masses, and coronary artery fistulas. Consequently, prompt diagnosis and surgical intervention are essential. We present a case of P-MAIVF secondary to infective endocarditis in a patient with a BAV, highlighting the rapidity of disease progression in this specific anatomy and underscoring the necessity of TEE when transthoracic findings are inconclusive.

## Case presentation (Table 1)

**Table 1:** Timeline of clinical course and management

Time point	Key clinical events and interventions
Onset of symptoms	Patient presented with fever and malaise. Initial labs showed neutrophilia. TTE suggested aortic valve malformation/regurgitation
Initial treatment phase	Empirical antibiotic therapy initiated. However, fever persisted (peak 39.4 °C) for two months
Referral and advanced diagnosis	Transferred to tertiary center. Blood cultures: Streptococcus haemolyticus. TEE & CT: Confirmed BAV and P-MAIVF communicating with LVOT
Preoperative planning	Multidisciplinary evaluation led to a surgical plan: aortic valve replacement (AVR) and repair of the mitral–aortic fibrous junction under extracorporeal circulation
Surgical intervention	Aortic valve replacement (Mechanical) + P-MAIVF repair (purse-string suture) under cardiopulmonary bypass
Postoperative follow-up	Uneventful recovery. No fever recurrence. Discharged with stable cardiac function

*Note:* AVR: Aortic Valve Replacement; BAV: Bicuspid Aortic Valve; CT: Computed Tomography; LVOT: Left Ventricular Outflow Tract; P-MAIVF: Pseudoaneurysm of the Mitral-Aortic Intervalvular Fibrosa; TEE: Transesophageal Echocardiography (or Echocardiogram); TTE: Transthoracic Echocardiography (or Echocardiogram)

### Initial presentation and early evaluation

A 28-year-old Han Chinese woman healthy Han Chinese woman presented with several days of fever and malaise and was admitted to a local hospital. Initial laboratory tests revealed elevated neutrophil counts. Transthoracic echocardiography suggested aortic valve involvement with suspected regurgitation. Empirical antibiotic therapy was initiated.

### Progression and referral

However, the patient experienced recurrent fevers over the subsequent two months, with temperatures peaking at 39.4 °C. Due to the persistent symptoms, she was transferred to our institution for further management. Blood cultures collected upon admission indicated a streptococcal bloodstream infection.

### Diagnostic imaging findings

**Fig. 1:**
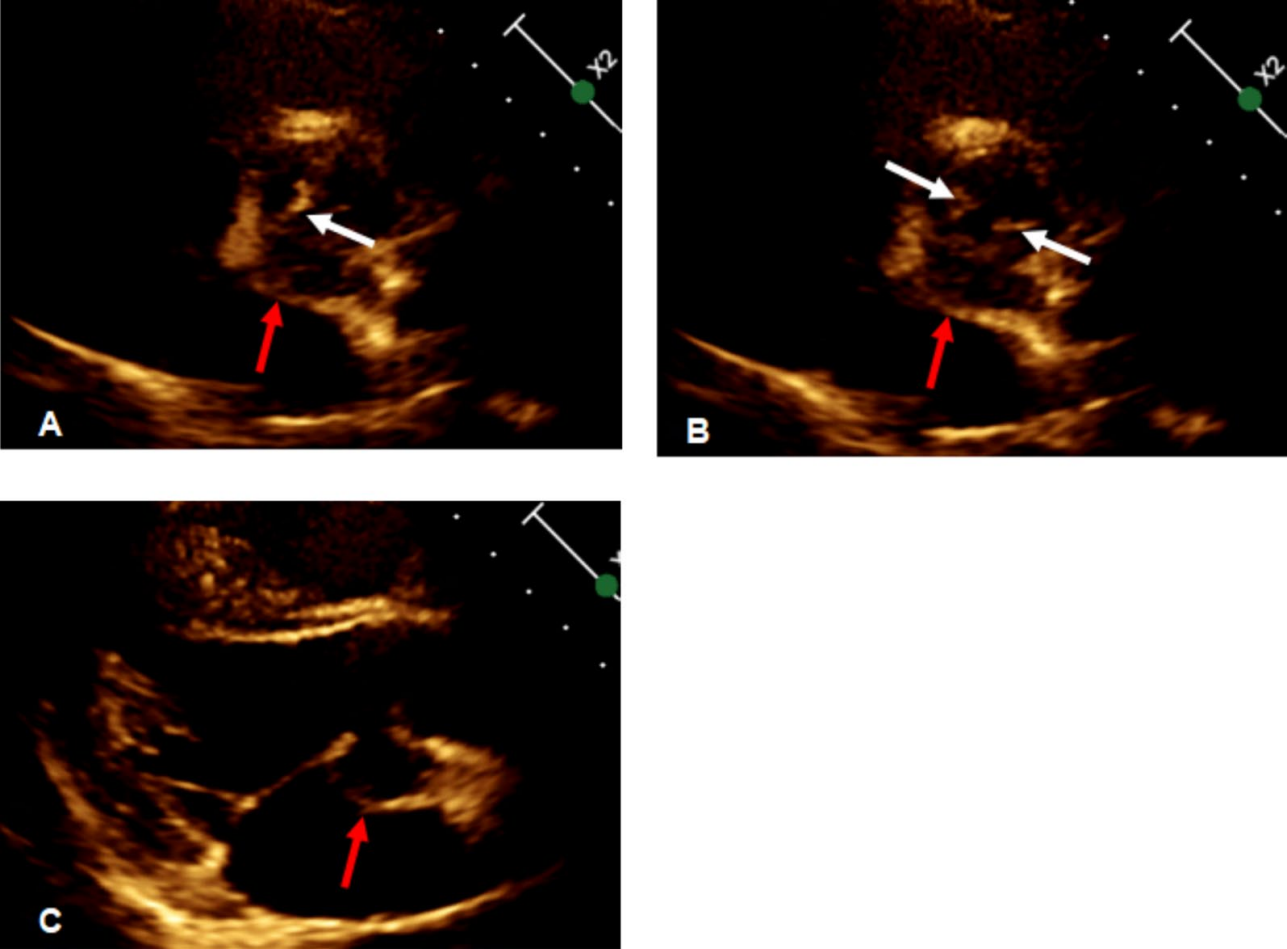
Transthoracic echocardiography (parasternal long-axis view). **A**, **B** Systolic and diastolic phases showing the bicuspid aortic valve. **C** An echolucent space (red arrow) is visible between the aortic annulus and the anterior mitral leaflet, suspicious for pseudoaneurysm

**Fig. 2:**
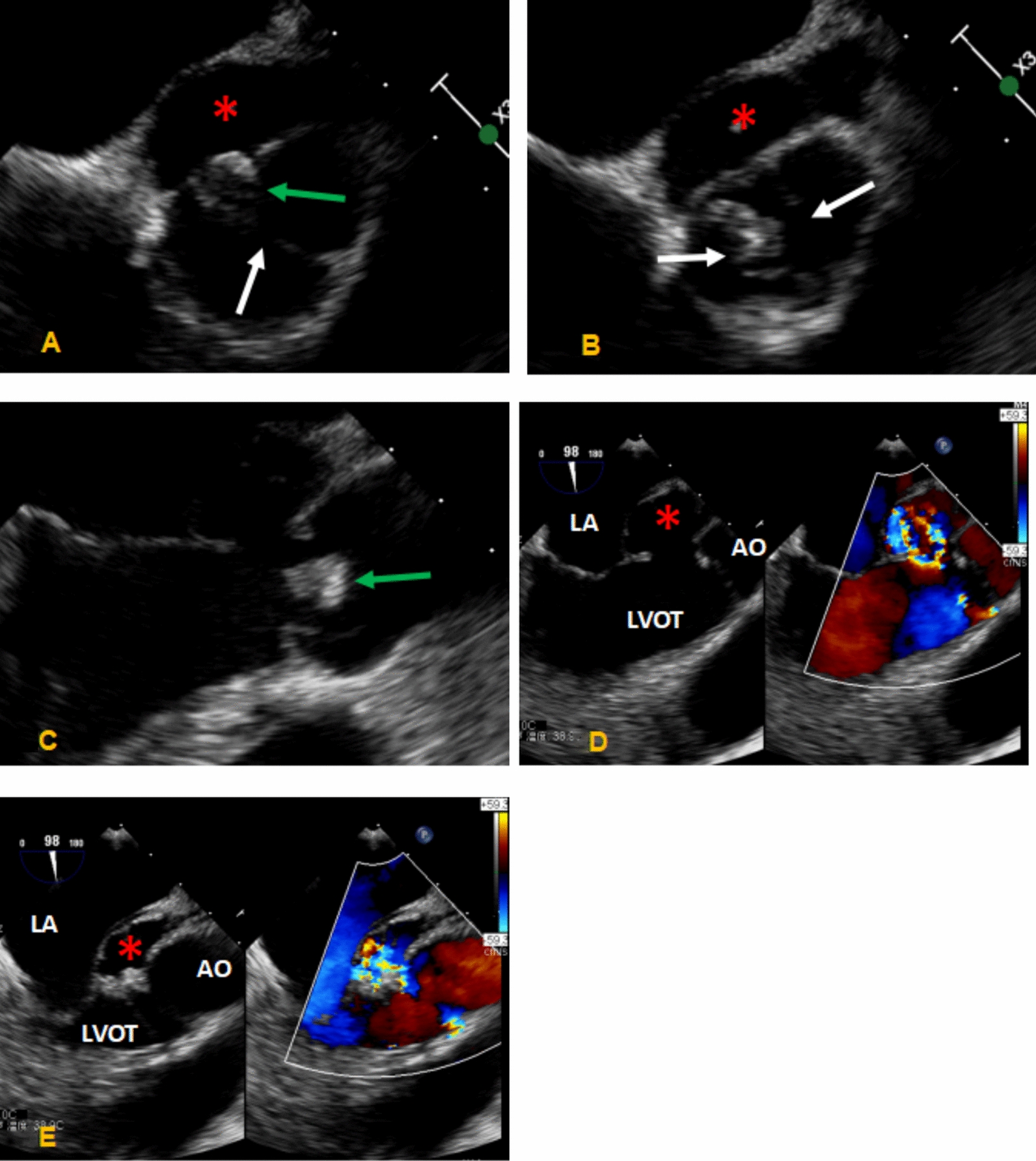
Transesophageal echocardiography. **A**, **B** Detailed view of the bicuspid valve showing "I" and "II" patterns during the cardiac cycle. **C** Vegetations on the leaflet (green arrow). **D** Systolic expansion of the pseudoaneurysm (asterisk) communicating with the LVOT. **E** Diastolic collapse of the cavity (asterisk)

**Fig. 3:**
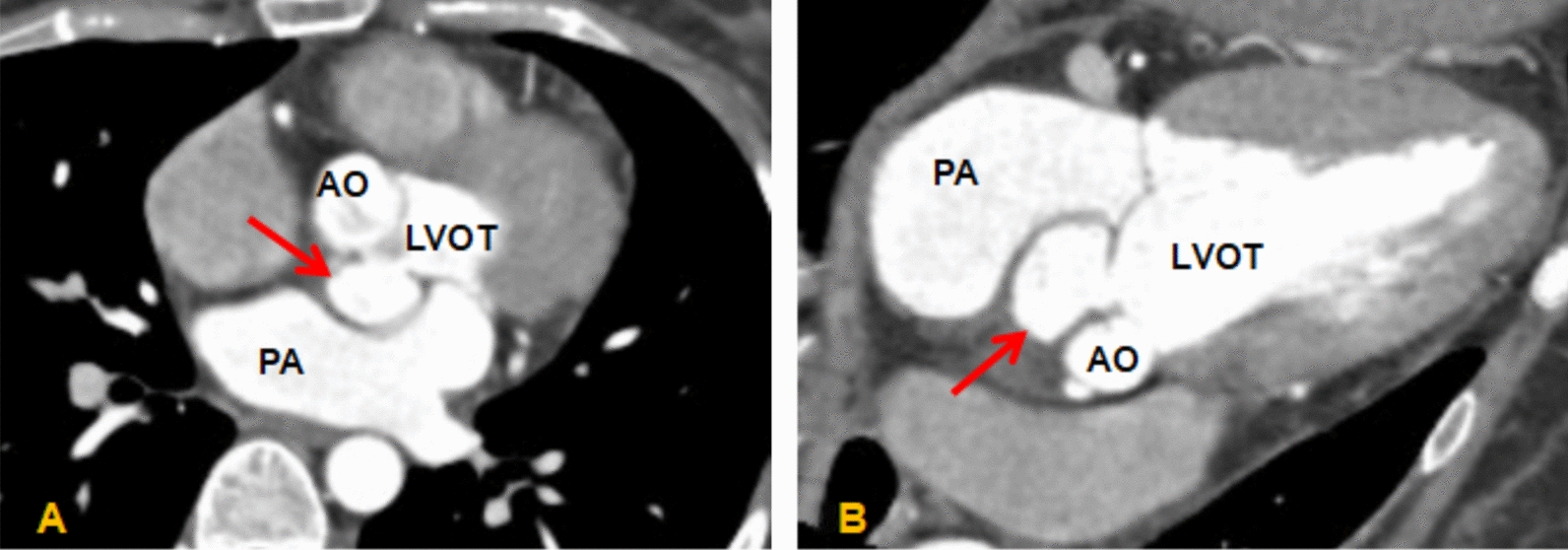
Contrast-enhanced CT. **A**, **B** Red arrows indicate the P-MAIVF cavity, confirming its communication with the left ventricular outflow tract (LVOT)

Comprehensive echocardiography was performed. Transthoracic echocardiography (Fig. 1) revealed a bicuspid aortic valve morphology and a cystic, echolucent area between the aortic annulus and the anterior mitral leaflet. Transesophageal echocardiography (Fig. 2) provided detailed visualization: the aortic valve exhibited a functionally asymmetric, diagonal orientation with restricted and abnormal leaflet motion. Both leaflets were thickened and bore mobile vegetations. A defect (approximately 7 mm in diameter) in the mitral–aortic intervalvular fibrosa was identified, with a pouch-like pseudoaneurysm arising posteriorly. This cavity communicated with the left ventricular outflow tract via the defect. Color Doppler imaging demonstrated bidirectional flow within the pseudoaneurysm (systolic inflow from the LVOT and diastolic outflow). Coronary computed tomography angiography (CTA) confirmed the presence of a pseudoaneurysm at the mitral–aortic intervalvular fibrosa (Fig. 3).

### Surgical findings and treatment

**Fig. 4:**
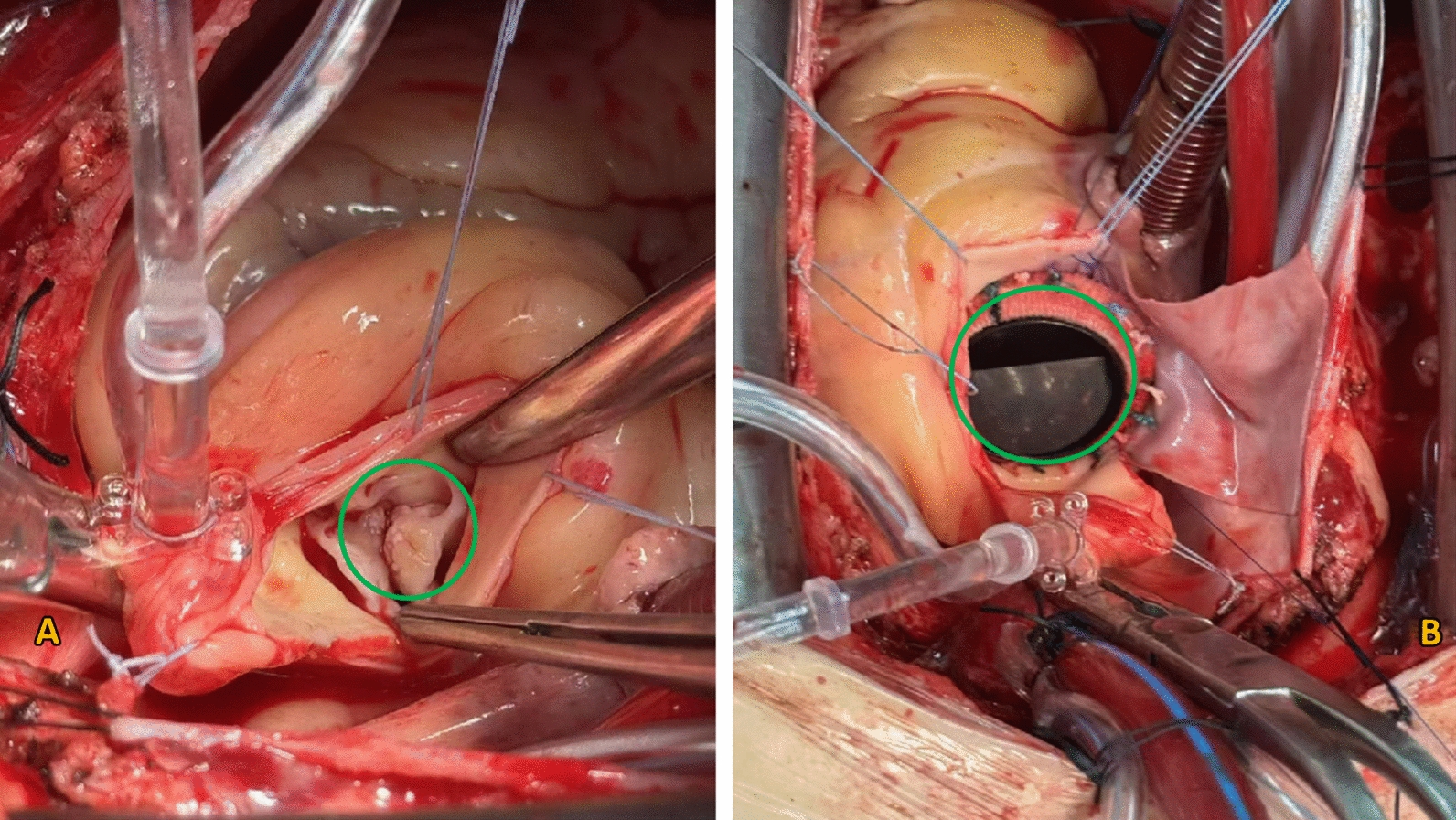
Intraoperative view. **A** Green circle highlights the vegetations on the aortic valve. **B** Green circle shows the implanted mechanical aortic valve

The patient underwent surgical intervention under cardiopulmonary bypass at the First Affiliated Hospital of Fujian Medical University. Intraoperative inspection confirmed a bicuspid aortic valve with multiple vegetations. Following aortic valve excision and partial debridement of the annulus, a rupture of the pseudoaneurysm into the left ventricular outflow tract was identified. The rupture site was closed with a purse-string suture. Subsequently, the aortic root was reconstructed using autologous pericardial patches, followed by implantation of a mechanical valve (Fig. 4). The total cardiopulmonary bypass time was 180 minutes.

### Postoperative outcome

The patient's postoperative course was uneventful. She recovered well, reported satisfaction with the care, and was compliant with the treatment plan. No fever recurred, and postoperative assessments showed preserved cardiac function.

## Discussion

### Anatomy and pathophysiology

The mitral–aortic intervalvular fibrosa (MAIVF) is a thin, avascular fibrous structure located between the left and right fibrous trigones of the cardiac skeleton, connecting the aortic valve's non-coronary cusp to the anterior leaflet of the mitral valve. This unique anatomy, coupled with exposure to high-velocity flow from the left ventricular outflow tract (LVOT), renders it susceptible to infection and mechanical stress. An infectious or inflammatory process can destroy this region, leading to the formation of a pseudoaneurysmal cavity that communicates with the LVOT, termed a pseudoaneurysm of the MAIVF (P-MAIVF) [1].

### Etiology and patient-specific risk factors

P-MAIVF primarily arises as a complication of infective endocarditis (IE), cardiac surgery, or trauma [2, 3]. Congenital structural weaknesses, such as those associated with a bicuspid aortic valve (BAV), are recognized predisposing factors [4].The regurgitant jet associated with BAV is typically eccentric, and this high-speed blood flow directly impacts the MAIVF area, making it more vulnerable to bacterial implantation (seeding) and mechanical damage, thereby forming a pseudoaneurysm. In the present case, the patient’s congenital BAV likely created an inherent vulnerability in the MAIVF region. Initially, echocardiography showed no vegetations, but after two months of irregular anti-infective therapy, significant vegetations and a well-formed P-MAIVF were evident, underscoring how delayed or suboptimal treatment in a susceptible host can facilitate rapid local destruction and pseudoaneurysm formation [5, 6].

### Clinical consequences and complications

The expansion of the pseudoaneurysm can compress adjacent structures (for example, left atrium, coronary arteries) or erode to form fistulae. Its rupture, either spontaneously or during surgery, can lead to cardiac tamponade, fistulization, or fatal hemorrhage [7, 8]. Therefore, early diagnosis and intervention are critical.

### Diagnostic features and differential diagnosis

The hallmark echocardiographic finding of P-MAIVF is a sac-like, echolucent cavity adjacent to the aortic root, showing systolic flow from the LVOT into the cavity and diastolic flow reversal on color Doppler [9, 10]. This case illustrates the diagnostic challenge: the P-MAIVF cavity and its characteristic flow can be mistaken for a perivalvular abscess with eccentric aortic regurgitation. Key differentiators include the abscess's more fixed morphology and the origin of regurgitant flow directly from the valve coaptation line. Differentiation from a sinus of Valsalva aneurysm is also crucial; the latter originates above the aortic annulus and typically ruptures into a right cardiac chamber [11, 12].

### Preferred imaging modalities

While transthoracic echocardiography (TTE) has limited sensitivity (~ 40%) for P-MAIVF, transesophageal echocardiography (TEE) is the cornerstone for diagnosis, with a sensitivity of 90–95% [13, 14]. In our patient, TEE precisely delineated the vegetation, the size and structure of the pseudoaneurysmal cavity, and the site of communication with the LVOT. Computed tomography and magnetic resonance imaging are valuable for assessing the relationship with extracardiac structures but are less suitable for dynamic assessment [15, 16]. Given its real-time capability, comprehensive hemodynamic evaluation, and suitability for follow-up, echocardiography remains the preferred initial and monitoring modality for P-MAIVF.

## Conclusion

This case illustrates the rapid progression from infective endocarditis to P-MAIVF in a patient with a predisposing bicuspid aortic valve, underscoring the critical need for early recognition and intervention. The favorable postoperative outcome highlights the effectiveness of timely surgical repair. For clinicians, this report emphasizes that in patients with congenital valvular anomalies presenting with persistent fever, a high index of suspicion and prompt, comprehensive echocardiographic evaluation—particularly with transesophageal echocardiography—are paramount to detect this serious complication. Early diagnosis, multidisciplinary collaboration, and definitive surgical management remain the cornerstone of preventing life-threatening sequelae and ensuring optimal patient outcomes.

## Data Availability

Availability of the data used and analyzed during the writing of the case report is under the responsibility of the corresponding author, and its distribution is authorized upon reasonable request.

## Abbreviations

P-MAIVF: Pseudoaneurysm of the mitral–aortic intervalvular fibrosa
TEE: Transesophageal echocardiography
BAV: Bicuspid aortic valve
MAIVF: Mitral–aortic intervalvular fibrosa
LVOT: Left ventricular outflow tract
